# Developing and Evaluating a Remote Quality Assurance System for Point-of-Care Ultrasound for an Internal Medicine Residency Global Health Track

**DOI:** 10.24908/pocus.v5i2.14433

**Published:** 2020-11-18

**Authors:** Steven Fox, Michelle Fleshner, Collin Flanagan, Thomas Robertson, Ayako Wendy Fujita, Divya Bhamidipati, Abdulrahman Sindi, Raghunandan Purushothaman, Thuy Bui

**Affiliations:** 1 Internal Medicine, University of Pittsburgh Medical Center, Presbyterian Pittsburgh, PA; 2 Allegheny General Hospital, Internal Medicine Pittsburgh, PA; 3 Department of Emergency Medicine, King Abdulaziz University Jeddah Saudi Arabia

**Keywords:** Point-of-Care Ultrasound, Quality Assurance, Global Health, Internal Medicine

## Abstract

**Background: **A quality assurance system is vital when using point-of-care ultrasound (POCUS) to ensure safe and effective ultrasound use. There are many barriers to implementing a quality assurance system including need for costly software, faculty time, and extra work to log images. **Methods: **With minimal funding or protected faculty time, we successfully developed an effective remote quality assurance system between residents rotating internationally and faculty in the US. **Results: **270 total exams were logged using this system (41 per resident over a 7 week period). Over the course of the implementation period, a significant increase was seen in average image quality (p = 0.030) and percent agreement with reviewer (p = 0.021). No significant increase was seen for percent images with quality rating 5/5 (p = 0.068) or for studies per resident per week (p = 0.30). **Discussion/Conclusions: **A quality assurance system for remote review and feedback of POCUS exams was successfully developed with limited available funding, using consumer-level software and an educational collaboration. Residents used the system regularly and demonstrated improvement in reviewer-rated image acquisition and interpretation skills. A similar system can be applied for physicians in any geographic area looking to learn POCUS, in partnership with local or international POCUS mentors. We detail a step-by-step approach, challenges encountered, and lessons learned, to help guide others seeking to implement similar programs.

## Background

Point of care ultrasound (POCUS) refers to the use of ultrasound by the treating provider at the bedside to answer focused clinical questions. The use of POCUS has become increasingly popular within the field of Internal Medicine (IM) worldwide 1, 2, 3, 4, 5. In settings where material and human resource constraints limit the availability of many diagnostic modalities, POCUS is a particularly valuable tool 4, 6, 7, 8. The increased utilization of POCUS demands a proportional commitment to creating structured curricula. Effective implementation of POCUS usage involves image acquisition, interpretation, clinical integration, and ongoing quality assurance (QA) 4, 9, 10, 11, 12. Many providers can obtain a foundation in image acquisition and interpretation through hands-on training, books, online videos, and e-learning. However, POCUS requires supervised learning and ongoing maintenance for competency, as many pitfalls and nuances exist to effectively use POCUS in the clinical setting 4, 13. Barriers to longitudinal education include time, access to expertise, and resource constraints 4, 14, 15, 16. Many educational leaders advocate prioritizing longitudinal and sustainable models for training 4, 17, 18. 

One potential means for ongoing POCUS education is a sustainable QA program. QA involves a peer-reviewed process where a POCUS expert evaluates a trainee’s ultrasound image acquisition and interpretation and provides feedback. This process is necessary for the development of competency 7, 9, 10, 17, 19, 20, 21. Portfolio development is an important component, as learners who develop an image portfolio demonstrate improved POCUS skills compared to those who do not 9.

Many challenges exist in implementing and maintaining a QA system, including institutional financial commitments to QA technology, protecting patients’ health information, and availability of experienced faculty. Additional challenges that may be more common in low-and-middle income countries (LMICs) include limited internet connectivity and infrastructure for data security 7, 22, 23. Proprietary software is often used to facilitate image storage, de-identification, transmission, and review, but this adds expense and training^14^, ^24^ and may have limited capability when working remotely. Currently there is no standardized structure for a QA system within Internal Medicine. Previously described ongoing remote QA systems involved intermittent (every 2 months) review sessions that required a trained provider to travel to the destination^7^ or had images sent via individual email 22. 

For this project we sought to establish a low-cost QA system for a US-based Internal Medicine residency/Global Health track to connect residents rotating internationally with a dedicated US-based POCUS QA faculty. This would serve to provide near real-time educational feedback, ensure safe and effective performance of POCUS studies, and provide a means for image portfolio development. Due to resource constraints, this needed to be done with minimal funding and faculty time. In this paper we describe a detailed step-by-step approach to implementing this low-cost, low-barrier remote QA system in a sustainable fashion, using consumer-level technology. We analyze the educational effectiveness of the system, and we share the lessons learned to help guide others implementing similar initiatives. 

## Methods

Residents in the Internal Medicine/Global Health track at the University of Pittsburgh Medical Center (Presbyterian/Shadyside) rotate at international sites as part of residency training. During these clinical rotations, POCUS is routinely used to aid in diagnosis. Prior training in POCUS included a 20-hour introductory didactic and hands-on training in image acquisition and interpretation, in topics including cardiac, lung, abdominal, and lower extremity DVT assessment. There was no formal system for image QA, and since this is such a critical component of a POCUS curriculum and implementation, this was identified as a key area for improvement. This project was initiated, developed, and implemented by a group of residents with involvement of faculty and program leadership in response to this educational need. It consisted of four phases: planning and preparation, developing the information/communication system, early implementation and promoting use, and evaluation/monitoring. The timeline, key components, and stakeholders for each phase are shown in Table 1. This methods section is subsequently organized by phase, and by each component within the phase. 

**Table 1:** Phases of Development and Maintenance of the Quality Assurance System

**Phase and Timeline**	**Key Components**	**Stakeholders**
**Planning and Preparation** *Starting 1 year prior to implementation*	Establishing goals and a shared mission Forming a coalition Obtaining permission of partner sites Ensuring access to ultrasound equipment Trialing different potential systems and workflows Enlisting QA faculty	Project leadership team Residency program leadership International partner site leadership QA faculty
**Developing the Information/** **Communication System** *6 months to immediately prior to implementation*	Determining plan for management of Protected Health Information Developing an image storage system Developing a Log and QA sheet Establishing workflows for image uploading Establishing workflows for image reviewing Developing a means for rapid communication within the group (WhatsApp group)	Project leadership team
**Early Implementation and Promoting use** *First few weeks of implementation*	Starting small, trialing the system, and scaling-up Orienting residents to the system Motivating residents to log images Motivating QA faculty to review images	Project leadership team All residents QA faculty
**Evaluation/Monitoring** *Ongoing*	Ongoing monitoring and system modification based on feedback Determining educational outcomes to assess	Project leadership team

### Phase 1: Planning and Preparation

#### Establishing goals and a shared mission

The goals of the project were determined by shared discussion among the resident and faculty leaders of the group, and included the following:

1. Provide educational quality assurance for POCUS during resident international rotations
2. Evaluate the utility and accuracy of resident-performed POCUS in this setting
3. Provide a means for quick clinical advice in challenging scenarios
4. Provide a means for POCUS portfolio development

#### Forming a coalition

The GH POCUS project was resident-initiated and implemented. A GH POCUS leadership team was formed including several Internal Medicine/Global Health residents and faculty, as well as fellows or faculty in other departments, including Emergency Medicine, Cardiology, and Critical Care Medicine. 

#### Obtaining permission of partner sites

Permission was obtained from department leadership at each partner site: Georgetown Public Hospital Corporation (Guyana) and Kamuzu Central Hospital (Malawi). The purpose of the project was explained, including the plan to exclude patient names and other identifiers from stored and transmitted scans. Feedback was solicited from these leaders to ensure functionality of the system in each location.

#### Trialing different potential systems and workflows

During the initial planning period a year prior to implementation, the leadership team trialed logging images using the available ultrasound equipment. This period was essential to identifying several key lessons learned, including needing to obtain a different ultrasound machine for the Malawi site, determining which information would be useful to include in the log, and successful image storing in Google Drive (Mountain View, CA, USA). 

#### Ensuring access to ultrasound equipment

The ultrasound machines used included a Phillips Lumify (Amsterdam, Netherlands), a GE Venue, and a GE V-scan (Boston, USA).

#### Enlisting Quality Assurance (QA) faculty

A question arose regarding who is eligible to review scans and provide feedback. In the absence of clear guidelines on credentialing for POCUS image QA within Internal Medicine, this was up to the judgment of the leadership team and the QA faculty. The QA faculty were attending physicians or fellows with substantial POCUS experience, including formal didactic training, regular use of POCUS for decision-making in clinical practice, and leadership in POCUS education and implementation in their institution. These criteria have been previously reported in the literature 25.

Members of the GH POCUS leadership team contacted prospective QA faculty, discussed the purpose of the project, the need for image QA, and what this would involve. QA faculty were told they would be contacted weekly with reminder emails and that they could review/QA as many studies as they felt able. This was entirely voluntary, with no financial incentives or protected time provided for image QA. 

The primary QA faculty consisted of three Internal Medicine attendings and one Emergency Medicine Ultrasound Fellow. Several additional faculty and fellows from cardiology and critical care were available for challenging cases, or to provide additional help as requested. 

### Phase 2: Developing the Information/Communication System

#### Determining plan for management of protected health information

It was of the utmost importance to ensure that patient privacy and confidentiality was not compromised in any way. As such, a unique image identifier number was assigned to each scan, and no identifying information was associated with the images at any point 26, 27. There was no coding done that would enable a third party to re-identify the data. 

Developing an image storage system (Google Drive)

The system had to be designed to accomplish the primary goals of the project in a way that had a minimal “added-work barrier” and was felt to be beneficial to all participants. The QA system was designed with three components: GH POCUS folder (in Google drive), Log and QA sheet (in Google drive), and a shared messaging group via WhatsApp (Menlo Park, CA, United States). 

#### Developing a Log and QA sheet

It was necessary to keep all the studies recorded in a single location. A single Log and QA sheet was developed as a Google sheet. This sheet served as a starting point for residents to record their case information and image interpretations, and as a starting point for QA faculty to perform image QA. See Table 2 for the list of data entered by the resident and by the QA faculty for each POCUS study in the Log and QA sheet. 

**Table 2:** Data Entries in Log and QA sheet

**User**	**Data Entries**
Image Uploader (resident)	· Unique Study ID · Type of Study · Country · One-liner (very brief clinical scenario - ex: middle aged male with fever and left leg swelling) · Primary Clinical Question · POCUS findings · Did POCUS answer your clinical question? (Yes, No) · Did POCUS change diagnosis? (Yes, No) · Did POCUS change management? (Yes, No) · Category (For Urgent QA, For non-urgent QA, No additional QA needed, poor quality images (do not QA), Educational Scan)
Image Reviewer (QA faculty)	· Based on the choice for “category”, a checkbox would automatically become checked in a column “Requesting Review”. · Reviewer Name · Reviewer Interpretation · Quality of Images (from 1 to 5, with anchors) · Agree with Interpretation (Yes, Yes with modifications, or No) · Feedback for Examiner

#### Establishing workflows for image uploading

A document was developed to describe the workflow for image uploading and was shared with the residents. This was helpful in clarifying the process and minimizing extra steps and would be useful when sharing the process with others. This document is included in the Supplementary Materials. 

#### Establishing workflows for image reviewing

A document was developed to describe the workflow for image reviewing, and was shared with QA faculty. This is included in the Supplementary Materials. 

#### Developing a means for rapid communication within the group (we used a WhatsApp group)

Though the primary purpose was educational QA, in practice, challenging cases arise in which residents and their patients could benefit from a more prompt expert review. We developed a WhatsApp group, including all resident and faculty members of the GH POCUS group, to help serve this purpose. This WhatsApp group was a shared message thread connecting all members of the GH POCUS group for quick communication, which was particularly useful for challenging cases and for learning points worth sharing. No patient identifying information was ever shared via this medium. 

With regard to resident supervision at the local site, Global Health residents are licensed in the country in which they rotate, and practice under direct supervision of physicians at that site. When QA faculty in the US are providing feedback on image interpretation and patient management, they are not considered to be supervising the resident or providing patient care. Rather, they are providing a remote physician-to-physician consultation, for advice only. Ultimately the decisions made are at the discretion of the physicians licensed in-country to provide patient care. In this way, remote QA faculty have no licensing requirement or liability related to this role. If POCUS interpretations are in doubt by the local physicians, they have the option to disregard and/or to obtain confirmatory imaging. 

**Figure 1:**
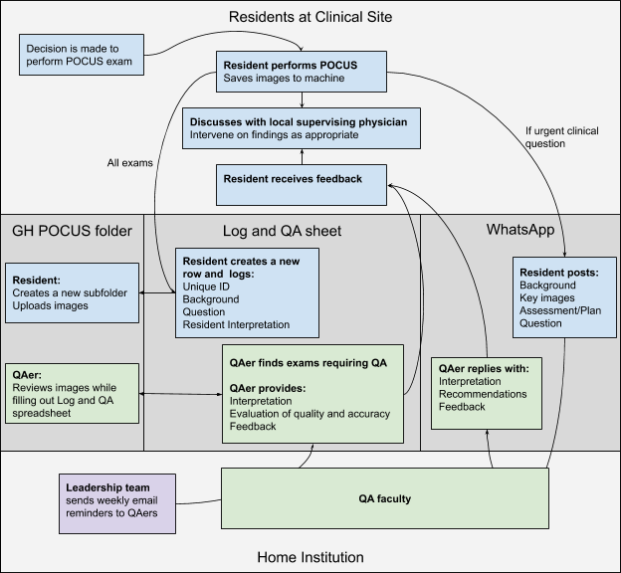
Process Map for the Quality Assurance System.

A process map for image for the information and communication system is shown in Figure 1. 

### Phase 3: Early Implementation and Promoting Use

#### Starting small, trialing the system, and scaling-up

The system was tested in progressively increasing capacity, starting with the leadership team with POCUS studies on 2-3 patients locally and progressively increasing to involve all residents using this system for all POCUS studies performed. 

#### Orienting residents to the system

The process of teaching the system involved several components. Prior to going abroad, all GH residents were invited to a dinner gathering where the process was introduced, along with a discussion of why this is important and a description of the steps. Once already abroad, the method was practiced by all residents in conjunction with the members of the leadership team who had worked to develop the system. There was a workflow document for reference, and the GH POCUS leadership team was available to answer questions and troubleshoot. 

#### Motivating residents to log images

The focus was not on making residents log images, but rather, in designing the system to naturally motivate image logging. A key framework for diffusion of change included the ideas of relative advantage, compatibility, complexity, trialability, and observability 28. It was important to inspire the idea that this is how to actually learn ultrasound, which will benefit residents’ patients and careers. Also critical was to minimize the “added-work barrier” by minimizing additional steps, making the system simple and intuitive, and integrating with existing workflows. Residents working together to complete the process and share cases with one another provided a sense of community, observability, and accountability. There also had to be immediate observable benefit to users. This could be fostered by having immediate feedback available through the WhatsApp group and by ensuring that the QA feedback provided was educationally beneficial. Examples of QA feedback are included in the Supplementary Materials.

#### Motivating QA faculty to review images

All QA faculty time was entirely voluntary, so QA faculty did the image review in their free time on top of their clinical duties. Similar as for residents, the focus was not on persuading faculty to QA many images. Rather, the focus was on creating an environment to foster regular QA by individuals with pre-existing intrinsic motivation and willingness to participate. There were many reasons for intrinsic motivation for QA faculty to be involved in this voluntary process including a desire to promote resident education, to promote a POCUS program and/or a GH program, and to gain personal POCUS and Global Health experience. The motivations can be grouped by the Volunteer Functions Inventory (VFI) 29.

Weekly reminder emails were sent to all QA faculty to prompt review, with direct links to image folders and the Log and QA Sheet along with a quick reminder of the workflow. Reviewers could do the image QA whenever was convenient for them, in whatever amount was feasible for them. 

### Phase 4: Monitoring and Evaluation

#### Ongoing monitoring, and system modification based on feedback

The development of this process required many iterations, and it was difficult to predict what would succeed and what would fail, so monitoring was important. In the early phases, this was largely practical/logistical troubleshooting, as described above in Early Implementation and Promoting Use. Key components to monitor after that included: ensuring 100% de-identification, ensuring consistency of image uploading and reviewing, reviewing QA feedback for themes, and seeking ongoing feedback on the process from residents and reviewers. As an example, through ongoing feedback from reviewers, it was recognized that labeling images with probe location/anatomic site would be helpful. Once that was identified, image labeling was encouraged. 

#### Determining educational outcomes to assess

We had two hypotheses to study: First, that this process is feasible, and second, that resident POCUS skills improve because of this process.

The specific outcomes to be measured were: number of studies logged each week per resident, percent agreement between residents and reviewers, average reviewer-rated image quality, and percent of POCUS studies given a 5/5 image quality rating (see Table 3 for the scales used). Each of these outcomes was determined on average over the course of the project. Additionally, each of the outcomes was determined for each week and regression analysis was performed. Bar graphs with trendlines were developed with week of implementation as the independent variable and the outcome as the dependent variable (y-axis). A trendline was created, and p-values for the trend were determined using regression analysis using the Microsoft Excel Data Analysis tool. The hypothesis was that each of the outcomes should increase over the course of the implementation.

**Table 3:** Image Quality and Agreement with Reviewer - The scale used for grading image quality and agreement with reviewer is shown. studies were considered highest quality only if they received a rating of 5. There was considered to be agreement only for “Yes” responses.

**Quality of Images**	**Do you agree with interpretation?**
1 - difficult to see anything much 2 - not great, but able to see some structures 3 - the image is ok - with substantial room for improvement 4 - the image is good but there is some room for improvement 5 - this is a great image	No Yes with modifications Yes

The implementation period was February 4, 2019 through March 23, 2019 (7 weeks). The first day of implementation was defined as the date the system was introduced to all the residents for use. The last day was defined as the date when the last resident returned from their international rotation.

Approval was obtained from the University of Pittsburgh Medical Center Institutional Review Board with educational exemption, IRB #PRO18040339. POCUS studies were being frequently performed already as part of routine medical care, and this system was designed to make the process safer, more effective, and more educational. All data was compiled as part of this process. No additional surveys or quizzes were applied. Since we studied data collected through this process of performing POCUS exams and QA, we were studying existing data so there was no recruitment of participants and obtaining informed consent was exempted. This was not considered human subjects research, as the research was studying an educational innovation, rather than studying individual patients or residents. Approval was also obtained from leadership at international partner sites.

## Results

**Figure 2:**
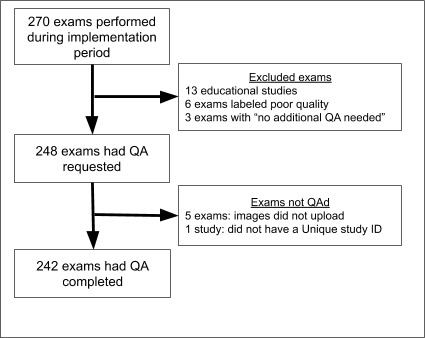
POCUS studies Logged and Quality-Assured

A total of 270 POCUS studies were logged over the 7 week period (see Figure 2). 248 studies had QA requested. The 22 studies that did not have QA requested were either because alternative QA was available or because the resident felt the images were too poor quality to warrant QA. 242 studies had QA completed (98% of all studies for which QA was requested). 

**Figure 3:**
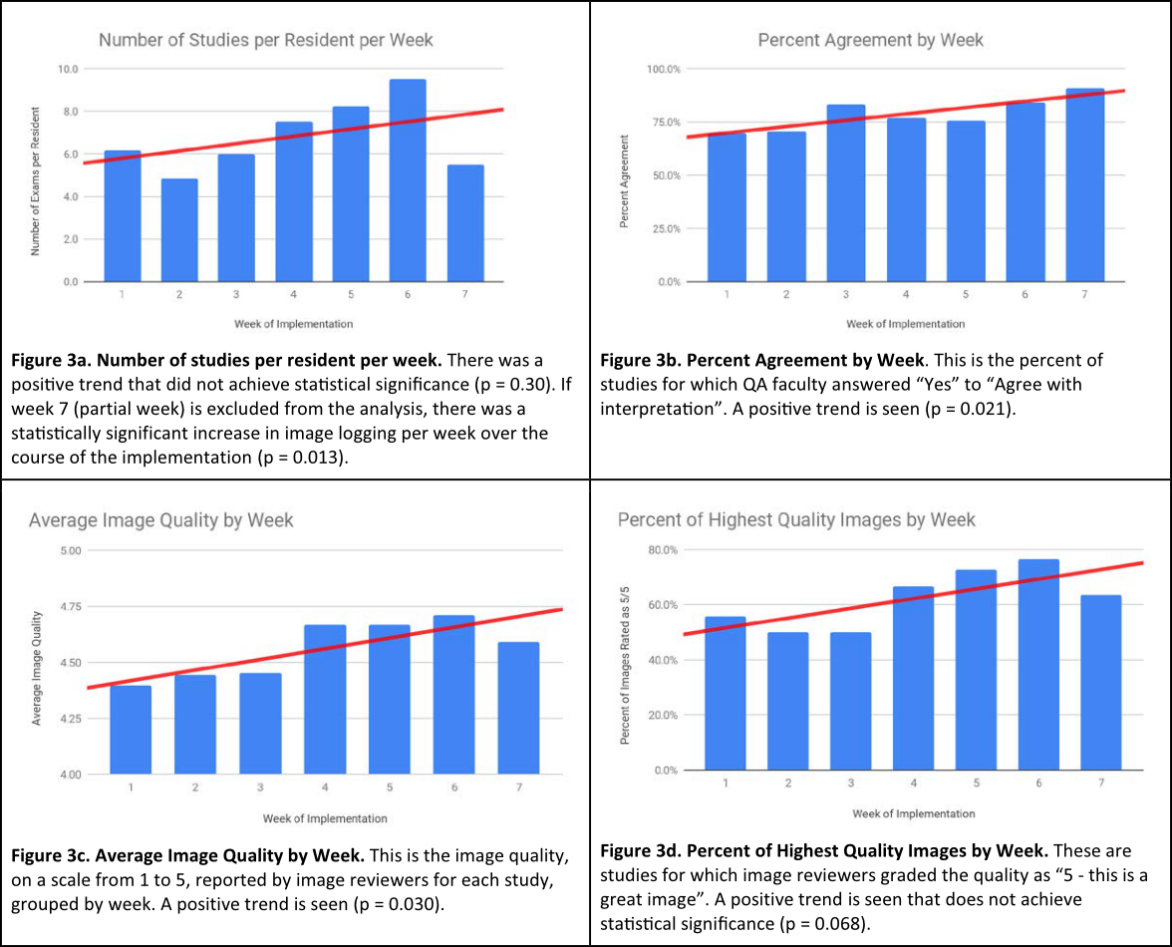
Educational Outcomes by Week

Residents completed an average of 41 POCUS studies each over the implementation period, at an average of 6.5 studies per week. 61.6% of interpretations were rated as the highest quality, 5/5. The average image acquisition quality rating for all POCUS studies was 4.55/5. Percent of interpretations that agreed with reviewer without modifications was 78.1%. Educational outcomes are shown in Table 4 and Figure 3. 

**Table 4:** Educational Outcomes by Week

**Week**	**Studies per resident per week**	**Percent Agreement with Reviewer**	**Average quality**	**Percent With Highest Quality**
1	6.1	69.8%	4.40	55.8%
2	4.9	70.6%	4.44	50.0%
3	6.0	83.3%	4.45	50.0%
4	7.5	76.7%	4.67	66.7%
5	8.3	75.8%	4.67	72.7%
6	9.5	84.2%	4.71	76.3%
7	5.5	90.9%	4.59	63.6%
**Average**	**6.5**	**78.1%**	**4.55**	**61.6%**
**P value for trend**	**0.30**	**0.021**	**0.03**	**0.068**

Average image quality increased significantly over the course of the implementation (p = 0.030). Percent agreement with reviewer increased significantly over the course of the implementation (p = 0.021). Percent images with quality rating 5/5 increased, but not statistically significantly (p = 0.068). studies per resident per week showed a non-significant increase (p = 0.30). Notably, week 7 was not a full week. If week 7 is excluded from the analysis, there was a statistically significant increase in image logging over the course of the implementation (p = 0.013). 

## Discussion

We describe a successful, low-cost, low-barrier, remote QA system between residents working internationally and dedicated multidisciplinary faculty support in the US. Using this system, residents demonstrated an objective, observed improvement in image acquisition and interpretation skills over a seven-week period. This is potentially applicable to other programs working to improve ongoing POCUS education in Internal Medicine as well as other specialties with residents locally or for those traveling to remote locations 9. This could also be applied more widely to any physicians working in locations where other providers with POCUS expertise are not available, to provide support for POCUS quality-assurance and feedback through a peer-to-peer image review network.

Several aspects of the project were key to its success. First was the formation of a coalition that included residents, faculty, and program leadership. Second was developing a system that naturally motivated its own use through integrating with workflows, minimizing extra steps, and providing quick feedback that created a sense of utility for users. Third was frequent troubleshooting and constant adaptation of the system to the practical setting. 

Some areas for improvement and next steps going forward include: measurement of turn-around-time for image review, feedback to image reviewers on the quality of the QA, having a dedicated ultrasound machine for this system at each site, and ongoing reevaluation of the system. In addition to being used for trainees traveling elsewhere, this system has the potential to be utilized as part of an initiative to teach POCUS for local trainees at international sites (and for any group of physicians seeking to learn POCUS in any geographic location) through a system of quality assurance by POCUS educators/mentors from either local and/or partnered institutions.

## Conclusions

A system for remote quality assurance of point of care ultrasound exams was successfully implemented to ensure effective use and to promote longitudinal training in POCUS, with limited funding using consumer-level technology. Residents demonstrated an increase in reviewer-reported image acquisition and interpretation skills through this process. This system has the potential to be applied for longitudinal POCUS training for trainees or groups of physicians in any geographic area, with local or international POCUS mentorship.

## Conflicts of Interest

None declared.

## Supplementary Material

Supplementary Documents Appendix 1 – Appendix 5.
