# Supplementary material for: Developing and Evaluating a Remote Quality Assurance System for Point-of-Care Ultrasound for an Internal Medicine Residency Global Health Track
Source: POCUS J. 2020 Nov 18;5(2):46–54. doi: 10.24908/pocus.v5i2.14433 (PMC9979927; doi:10.24908/pocus.v5i2.14433)
Supplement: Supplementary Documents [file pocusj-05-14433-s001.pdf]

Supplementary Material (Appendix)

Appendix 1. Template Log and QA sheet

Steps

1. Examiner fills out grey and yellow portion during or soon after performing POCUS
2. Reviewer fills out the blue portion when convenient

Screenshots

Log and QA sheet

	A	B	C	D	E	F	G	H	I	J	K	L	M	N	O	P	Q	R
1	Study Info	POCUS Log									Reviewer 1 QA						Self-QA (if applicable)	
2	Unique Study ID:	Type of Study	Location	One Liner	Primary Clinical Question	Summarize your findings	POCUS changed Dx	Did POCUS answer your clinical question?	POCUS changed mgmt?	Category	Requesting review?	Reviewer Name	Reviewer Interpretation	Quality of Images	agree w/ interpretation?	Feedback for examiner	Type of Study	Interpretation
3	ABCMMDD01	Lung	Malawi	middle aged m with dyspnea	A-lines/B-lines /Consolidation	A profile, no b lines, effusion, or consolidation noted	No	Yes	No	For non-urgent QA	☑	QED	A line profile. No B lines or pleural effusion.	4 - the image is good but there is some room for improvement	Yes	Could also comment on presence of lung sliding		
4											☐							
5											☐							
6											☐							
7											☐							
8											☐							
9											☐							
10											☐							
11											☐							
12											☐							
13											☐							
14											☐							
15											☐							
16											☐							
17											☐							
18											☐							
19											☐							
20											☐							
21											☐							
22											☐							
23											☐							
24											☐							
25											☐							
26											☐							
27											☐							
28											☐							
29											☐							
30											☐							
31											☐							
32											☐							

b) Options for entries (using data validation)

	A	B	C	D	E	F	G	H	I	J	K	L	M	N	O	P
1	Study Info	POCUS Log										QA				
2	Unique Study ID:	Type of Study	Location	Pre-POCUS diagnosis	Clinical Question	Summarize your findings	Post-POCUS Diagnosis	Is your Post-POCUS diagnosis the same as your Pre-POCUS diagnosis?	Did POCUS answer your clinical question?	Did POCUS change your management?	Category	Reviewer Name	Reviewer Interpretation	Quality of Images	Do you agree with interpretation?	Feedback for examiner
3		Echo	Malawi		LV function			Yes	Yes	Yes	For urgent QA			1 - difficult to see anything much	Yes	
4		Lung	Guyana		Pericardial effusion			No	No	No	For non-urgent QA			2 - not great, but able to see some structures	Yes with modifications	
5		Abdominal	Birmingham		RV function						No additional QA needed			3 - the image is ok - with substantial room for improvement	No	
6		Cardiopulmonary Combined	(if other, type here)		Pleural effusion						Poor quality images - do not QA			4 - the image is good but there is some room for improvement		
7		Procedural			A-lines/B-lines/Consolidation						Educational scan			5 - this is a great image		
8		MSK/Skin and Soft Tissue			Evidence of cirrhosis											
9		Vascular			Evidence of abdominal TB											
10		Renal			Hydronephrosis											
11		(if other, type here)			Assess bladder or foley											
12					Splenomegaly (or other spleen evaluation)											
13					Ascites											
14					DVT											
15					Evidence of abdominal free fluid or air											
16					Evaluate for malignancy											
17					Further characterize mass											
18					Lung sliding											
19					Volume status											
20					Kidney or liver as etiology of volume status?											
21					Kidney size / Evidence of CKD											
22					Gallbladder pathology											
23					(if other, type here)											
24																
25																
26																
27																
28																
29																
30																
31																
32																

Appendix 2. Workflow for Imaging Reviewing

Workflow for Imaging Reviewing

1. Open "Log and QA" sheet: (Link included)
    a. Go to the "Reviewer 1 QA" section (Column J)
    b. Studies with the box checked under "Requesting Review" do require QA (if the box is not checked, you can skip)
2. Open "GH POCUS" folder (you should have access to this) → the folder for the individual resident (based on the initials)
    a. (Link included)
    b. Open the folder whose name matches the "Unique Study ID" in column A of the Log and QA sheet
    c. Review images
3. Fill out blue "Reviewer 1 QA" section (Columns J-O) in "Log and QA" sheet

Then move on to next study

Tips on providing your interpretation

- Focus on specific questions that can be answered on POCUS.
- Be appropriately specific in interpretation for scope (ex: better to say "hypoechoic kidney lesion" than "benign appearing renal cyst")
- Helpful to comment on integration of findings here as well (ex: no DVT seen here, but the patient has a high-pre-test probability, so DVT is not ruled out)

Tips on providing feedback for examiner

- Be specific when commenting why image quality is not optimum
- Provide advice on how image quality could be improved (ex: when looking for B lines, should increase depth to at least 16 cm)
- Can include helpful literature or evidence-based learning points here

Notifications

- You should receive an email weekly to request that you QA images (you are welcome to QA images more frequently than that)
- For urgent QA needs, there will be a message on the whatsapp group
- If you would like to provide additional direct feedback to the resident, you may optionally email them directly

Other notes

- More than 1 person may QA an image. If you are the second person, use the farther right blue sections for additional reviews.
- If you are annoyed by the WhatsApp messages, you can silence notifications for that chat
- If at any point you decide you don't want to QA images anymore, please let us know

Contact (email provided) with any questions

Appendix 3. Overview of Workflow for Image Uploading

**Before beginning**
Ensure you are a member of/are shared on<br>● The UPMC Global Health google drive folder<br>● The GH WhatsApp group
If you will be using your phone, the following apps may be helpful<br>● Google Drive<br>● Google sheets<br>● WhatsApp

**For each study**
**Overall workflow**<br>1) Obtain images<br>2) Move images to google drive folder (see separate site and machine specific workflows)<br>3) Post in WhatsApp if urgent review would be helpful<br>4) Fill out *POCUS Log* section in *Log and QA sheet*
<u>Tips for recording images</u>:<br>● Obtain multiple views<br>● Remember to optimize the image (depth, gain, etc…)<br>● Record video for 5-10 seconds (for lumify, set clip duration to 10 seconds)<br>● Use color flow if necessary<br>● Perform measurement or other annotations on still images as needed<br>● Label images as able
<u>Using the Log and QA sheet</u>:<br>● Enter Log and QA sheet within GH POCUS folder<br>● Create a new row, starting with Unique Study ID<br>● Input remaining cells in "Case info" and "POCUS Log" section.<br>● Create separate lines for multiple studies (ex: cardiac and abdominal)<br>● If multiple clinical questions within a single line, choose the "Primary" clinical question<br>● Be sure to choose image category (ex: educational scan, urgent QA needed, etc...), which will help determine if QA is needed or not

Check back on the log intermittently to review your images that have been QAd and to read the feedback

Contact (email provided) with any questions

Appendix 4. Machine-Specific Workflows for Image Uploading

<u>Uploading Images from V-scan</u>

**For each study**
Obtain images and save to machine<br>● Clips are obtained by pressing disk icon in lower right. These are retrospective.<br>● Still images are obtained by freezing (center white button), then pressing disk icon in lower right
Move images to google drive<br>● Attach V-scan to dock, then connect USB cable to your computer<br>● Move images to your computer<br>○ Find your image folder in the external disk, which should appear under file explorer, finder, or equivalent. Move it to your computer<br>○ Rename folder with Unique study ID<br>○ Remove unacceptable images<br>○ Try to name individual images with anatomic site (ex: R Kidney)<br>● Move images to drive<br>○ In drive, enter your Images Folder and select "Folder Upload" and find the above folder<br>○ Can alternatively choose folder upload directly from the V scan external disk folder
Fill out Log and QA sheet

Uploading Images from Lumify

**Before beginning**
Create a folder for yourself on the tablet (note this will depend on tablet used)<br>● Go to *Files*<br>● Select *Internal Storage* under *Local*<br>● Select *Lumify*<br>● Create a folder with your name
In Lumify app, create an *Export Destination* with your name<br>● Open Lumify App and go to *Export Destination*<br>● Click *Add new* and set the nickname to your name<br>● Select *local directory or media*<br>● Modify directory to be the same name as folder created above (should be *Lumify/"Your Name")*<br>● Click Save

**For each study**
Obtain images and save to machine<br>● Under patient last name box, enter the unique study ID<br>● Click start exam and begin scanning<br>● Hit the record icon to begin recording clip. Hit it again to stop recording<br>● Hit the camera icon to record a still image.<br>● Once you are finished, click "end exam"
Move images to google drive<br>● Attach V-scan to dock, then connect USB cable to your computer<br>● Move images to your computer<br>○ In Lumify app - locate your images under "Saved studies" - title should be unique ID number<br>○ Click on your unique ID number and hold until it turns orange<br>○ Click "export" on the top right corner and select your name<br>○ Now, your images will be saved on the tablet<br>● Move images to drive<br>○ Go to "files" on the HuaWei tablet<br>○ Locate the folder pertaining to each unique ID number and open it<br>○ Select all files you would like to upload<br>○ Click "more" and select "share"<br>○ Select "save to drive"<br>○ Click on the section labeled "folder" and create a folder with your unique ID number within the folder for your name<br>○ Click select and save<br>Now your images should be in your folder within the GH POCUS folder, which can be accessed by QA faculty
Fill out Log and QA sheet

Appendix 5. Examples of QA Feedback for residents

**Feedback**
“Moving the probe rapidly makes it difficult to interpret lung US, especially B-lines. Remember that one of the diagnostic criteria with B-lines is that it moves with the pleura. If you think you see B-lines, hold the probe still and ask the patient to breathe.”
“great images, on the renal scan if you're concerned, try to get other axis with careful fanning, and when facing anechoic structure around the renal pelvis (like one of the images), try to put color on it to see if its static> hydro, or glowing> renal vasculature
“I actually thought there were B lines all throughout. They were just confluent so they didn't look like typical b lines. But confluent B lines are often more significant than separate isolated B lines. Also, should increase depth to 16 cm when looking for B lines, these clips had depth of 12 cm.“
“RUQ image seems to be reversed from conventional probe orientation i.e. probe marker between superior and toward pt right”
“nicely done, especially showing the saphenofemoral junction compression test on both sides and then going distally from there”
“GREAT lung ultrasound. Seemed from description this was all left lung? For clarifying static vs dynamic air b'grams, I'd advise holding the probe stationary when you have a nice view of them, and seeing if they slide up and down within the bronchi with the respiratory cycle. You obtain a great view of the air bronchograms in the second image, and there is some movement within the bronchi, but difficult to tell if due to respiration or probe movement. In the original study dynamic air bronchograms were always PNA, but Static air bronchograms were PNA 50% and atelectasis 50%. Thus useful if dynamic, not so useful if static.”
“Note the small amount of fluid above the diaphragm on the right. If you want to be sure that there is no pleural effusion, you should have the spine, the diaphragm and the kidney in the same image. That is the correct plane to r/o pleural effusions, in other planes, you might miss a small effusion.”
“If any way to label or title images to know where on lung/what side they're from would be helpful!”
